# Accuracy of femoral head center reconstruction using a straight cementless rectangular stem: An *in-silico* study comparing elderly and middle-aged patients

**DOI:** 10.1016/j.jor.2025.07.017

**Published:** 2025-07-23

**Authors:** Hidde D. Veldman, Ide C. Heyligers, Philip C. Noble, Tim A.E.J. Boymans

**Affiliations:** a Department of Orthopaedic Surgery and Traumatology, Zuyderland Medical Center, H. Dunantstraat 5, NL-6419 PC, Heerlen, the Netherlands; b Department of Orthopaedic Surgery, Maastricht University Medical Center+, P. Debyelaan 25, NL-6229 HX, Maastricht, the Netherlands; c School of Health Professions Education, Maastricht University, P.O. Box 616, NL-6200 MD, Maastricht, the Netherlands; d Center for Orthopedic Research, Innovation & Training, Department of Orthopaedic Surgery, University of Texas, 5410 W Loop South, Suite 1300, Bellaire, TX, 77401, United States of America

**Keywords:** Total hip arthroplasty, Femoral head center, Very elderly, Templating, Proximal femur morphology, Cementless stem

## Abstract

**Introduction:**

Accurate reconstruction of the femoral head center (FHC) is essential for restoring hip biomechanics in total hip arthroplasty (THA). Previously described age-related morphological changes—such as canal widening and a mediocaudal FHC shift—may complicate anatomical reconstruction in all age-categories using a single non-modular cementless stem. This study assessed the capacity of such implant to achieve adequate FHC reconstruction across age groups and sexes.

**Methods:**

Virtual implantation of a non-modular cementless stem (SL-PLUS™) was performed in CT-based 3D reconstructions of 148 femora from middle-aged (<80 years) and very elderly (≥80 years) subjects. For each case, the optimal implant size, type (standard or lateral), and modular head (-4 mm, 0 mm or +4 mm) were selected. FHC deviation was measured in three dimensions; reconstructions were considered adequate if < 5 mm in all directions.

**Results:**

Overall, 92.4 % of reconstructions were considered adequate. No significant differences in reconstruction accuracy could be detected between age or sex groups. Very elderly males required significantly larger stem sizes than middle-aged males (mean size 6.4 vs. 5.1; p < 0.001). Lateralized stems were used more frequently in very elderly males (76.0 %) than in middle-aged males (44.4 %; p = 0.001). Reconstruction failure occurred in 11 cases, mostly due to a reduced mediolateral offset in femora with high native ML-offsets and/or low neck-shaft angles (8 out of 11 cases).

**Conclusions:**

A single non-modular cementless stem enables satisfactory FHC reconstruction in most patients, regardless of age or sex. However, certain anatomical configurations may exceed its reconstructive capacity. Careful preoperative planning is essential to identify cases that may need an alternative approach.

## 1 Introduction

Total hip arthroplasty (THA) is the definitive treatment for end-stage hip osteoarthritis. A key surgical objective is the accurate restoration of the femoral head center (FHC) relative to the rest of the femur and thus the femoral attachment sites of the hip musculature. Adequate FHC restoration is essential to optimize postoperative function and reduce the risk of complications, including leg length discrepancy, joint instability and dislocation, abductor deficiency, gait disturbances, limited range of motion (ROM), impingement, accelerated wear, and overall patient dissatisfaction.^1^, ^2^, ^3^, ^4^, ^5^, ^6^, ^7^, ^8^, ^9^

Anatomical studies have revealed an age-related shift of the FHC towards a relative varus position; described by a decrease in neck-shaft angle (NSA) and femoral head height, and an increase in mediolateral offset (ML-offset) with increasing age.^10^, ^11^, ^12^, ^13^, ^14^ The femoral canal is subject to age-related changes as well.^11^^,^^12^^,^^15^, ^16^, ^17^ With advancing age, progressive cortical thinning leads to widening of the medullary canal and a reduction in meta-diaphyseal taper. This change in canal shape is more profound in (postmenopausal) females and leads to the implantation of larger cementless stems in order to achieve durable primary stability through the mechanical interlock developed between the surface of the stem and the endosteal surface of the canal.^18^, ^19^, ^20^, ^21^

As femoral canal geometry dictates stem size and position, it directly influences the reconstruction of external femoral anatomy in non-modular stem designs. A wide variety of cementless non-modular stems is available today, most of which offer lateralized options and/or modular heads for fine-tuning. However, these designs remain constrained by the configurations provided within each manufacturer's portfolio. Considering the morphological changes associated with aging, and the lack of a consistent correlation between internal canal geometry and external proximal femoral morphology in the elderly,^22^ the question arises whether current non-modular cementless stem designs can equally reconstruct external femoral anatomy across the different age groups typically indicated for THA. Although multiple studies have evaluated the effectiveness of various stem designs in restoring native hip anatomy,^4^^,^^6^^,^^23^, ^24^, ^25^, ^26^, ^27^, ^28^, ^29^ the impact of age-related changes in femoral morphology on the accuracy of anatomical reconstruction remains unexplored.

This study aimed to assess the ability of a single cementless non-modular stem design to achieve anatomical FHC reconstruction in very elderly (≥80 years) versus middle-aged (<80 years) individuals, and to identify failure modes in inadequately reconstructed cases. We hypothesized that: (1) very elderly patients require larger stem sizes due to age-related widening of the femoral canal; (2) the increased-offset variant of the investigated stem may improve reconstruction accuracy in elderly subjects by accommodating the age-related medial shift of the FHC; and (3) age- and sex-related variations in femoral morphology may limit the ability of a single stem design to consistently restore native anatomy across the demographic groups included in this study.

## 2 Methods

### 2.1 Design

A cross-sectional study was conducted using three-dimensional (3D) computer models of healthy femora.

### 2.2 Subjects

Femora from two groups were included:

1. Very elderly group: 90 Caucasian subjects (50 males, 40 females) aged ≥80 years (mean 84, range 80–105). Femoral CT scans were acquired for diagnostic purposes unrelated to the hip, using a Sensation Open scanner (Siemens, Erlangen, Germany; 500 mm field of view, 1 mm slice thickness). Each image consisted of 512 × 512 pixels (0.98 mm resolution). The local institutional review board approved this study (07-T-44/IIIb) and informed consent was obtained from all patients.
2. Middle-aged group: 58 Caucasian cadavers (54 males, 4 females) aged <80 years (mean 52, range 20–79). Post-mortem CT scans were acquired using a LightSpeed scanner (GE Medical Systems, Chicago, IL; 384 mm field of view, 0.625 mm slice thickness). Each image consisted of 512 × 512 pixels (0.75 mm resolution). The dataset was obtained from the Institute of Orthopaedic Research & Education, Houston, USA.

All subjects in both groups with evidence of previous trauma, surgery, or bony pathology (malignance or metabolic disease) were excluded.

### 2.3 Three-dimensional reconstruction

Cortical bone was segmented in Mimics v10.01 (Materialise, Leuven, Belgium) based on pixel radiodensity (Hounsfield units, HU). Following Rathnayaka et al.,^30^ each femur was subdivided into four regions, each assigned a region-specific minimum HU threshold to optimize cortical bone selection: (1) the head/neck region, extending to 1 mm below the femoral head, was segmented with a threshold of ≥226 HU; (2) the proximal metaphysis, defined as the region up to 30 mm below the lesser trochanter, was segmented using the patient-specific 50 % threshold method described by Hangartner et al.^31^; the diaphysis, extending to 20 mm proximal to the patella, used a threshold of ≥662 HU; and (4) the distal femur, segmented with a threshold of ≥226 HU. All segments were subsequently merged into a single solid femur model and imported into a computer-aided design (CAD) environment (Rapidform 2006, Inus Technology, Rock Hill, SC) for landmark identification, virtual implantation, and outcome measurements.

### 2.4 Anatomical landmark identification

A best-fit sphere was placed on the femoral head; its geometric center defined the FHC. An XYZ coordinate system was established with the FHC as origin, based on International Society of Biomechanics guidelines.^32^ The center of the knee (CoK) was the midpoint between the medial and lateral epicondyles. The mechanical (Y-) axis connected the FHC and CoK. The Z-axis (mediolateral) was perpendicular to the Y-axis in the plane connecting the medial and lateral epicondyles with the FHC. The X-axis (anteroposterior) was orthogonal to both.

The proximal femur axis (PFA) and the femoral neck axis (FNA) were derived by circumscribing the cortical bone with circles and connecting the centroids of these circles, as described in detail previously.^10^ PFA construction was based on the work of Maruyama et al.^33^ and focused on the proximal region extending from 25 % to 35 % of the total femur length. The methodology for constructing the femoral neck axis (FNA) was based on the work of Sugano et al.^34^ The NSA was defined as the angle between the PFA and the FNA in a coronal plane and the ML-offset is the mediolateral distance between the PFA and the FHC. The femoral neck anteversion angle (FNAA) was defined as the angle between the FNA and the ‘table-top plane’, which is the plane simulating the orientation of the femur lying on a flat table.

### 2.5 Femoral component model

**Fig. 1:**
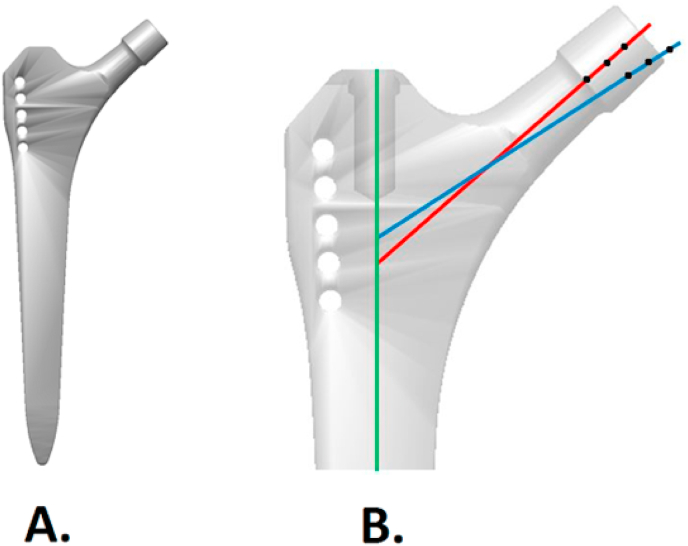
Femoral component design used for this study. (A) The SL-PLUS™ stem (Smith & Nephew, London, UK) is a straight, dual-tapered stem with rectangular cross-section and is available in 14 subsequent sizes. (B) For each stem size, two stem types are available with different caput-collum-diaphyseal (CCD) angles, but equal neck height: (i) the standard stem (131° between stem axis [green] and neck axis [red]) and (ii) the lateralized stem (123° between stem axis [green] and neck axis [blue]). Head position could also be simulated by using either a -4mm, 0 mm or +4 mm head, thus resulting in 6 possible femoral head center locations per stem size.

The SL-PLUS™ stem (Smith & Nephew, London, UK) was selected for this study. This common design is considered the third generation of the original Zweymüller stem and has excellent clinical results, also in very elderly subjects with femora of cylindrical (“stove-pipe”) morphology.^35^, ^36^, ^37^ Reverse-engineered 3D CAD models of the stem were created. Each stem size was available in two caput-collum-diaphyseal (CCD) angles—131° (standard) and 123° (lateralized)—and permitted head modularity with three correction options (−4 mm, 0 mm, +4 mm), resulting in six possible FHC positions per stem size (Fig. 1).

### 2.6 Virtual implantation procedure

The investigated stem is a straight, double-tapered, diaphyseal-fitting stem that follows the 'fit without fill' principle, relying on 3-point cortical contact for fixation. The stem gains axial stability through the double taper and rotational stability through the rectangular cross section.^38^

The stem was virtually implanted according to the implant's principles. Initially, the stem was implanted with the stem axis aligned to the PFA. Subsequently, slight adjustments were made in both the coronal and sagittal plane, aiming alignment between stem and the trajectory of the endomedullary canal, and optimizing the fit against the endosteal surface. We aimed to maximize contact between the corners of the stem and the cortex of the femur and thus positioned the stem as deep as possible into the femoral canal without ‘overbroaching’ the femoral canal. Previous studies that performed a virtual implantation technique of cementless femoral components aimed to obtain cortical bone contact without cortical penetration.^27^^,^^39^ However, since it is known that the corners of the studied implant cut grooves in the cortical bone,^38^ we allowed some cortical penetration to occur. A maximum of 1 mm cortical penetration on opposite sides was chosen, which is comparable with the maximum of 0.8 mm cortical penetration that was considered realistic in a previous finite element study.^40^ The cortical penetration on opposite sides was measured on cross-sections along the full length of the stem.

If the FHC of the femoral component was located proximal or distal to the native FHC (FHC_native_), a smaller or larger sized implant was loaded into the model respectively. The stem/head combination that reconstructed the FHC_native_ most closely with the required fixation principles, was considered FHC_reconstructed_. The implantation procedure was performed by an orthopaedic surgery resident (HV) under the supervision of an orthopaedic hip surgeon (TB), both of whom were experienced with the software used. The refinement process was conducted collaboratively, with adjustments made until consensus was reached on the final positioning of the stem. In complex cases, a third author (IH), a senior hip surgeon, was consulted to ensure optimal implant positioning.

### 2.7 Outcome parameters

The main outcome was the absolute distance between FHC_reconstructed_ and FHC_native_. Subsequently, the deviation was analysed per dimension (superior–inferior, mediolateral, anteroposterior). Deviation thresholds were set at <2.5, <5, <10, and ≥10 mm based on clinical relevance,^6^, ^7^, ^8^, ^9^ ≥5 mm deviation in in any dimension was considered inadequate. NSA and FNAA were re-measured post-implantation using the stem's neck axis, representing the postoperative values.

### 2.8 Statistical analysis

Means, standard deviations, and ranges were calculated for all variables. Normality was assessed using the Shapiro–Wilk test, and equality of variances was tested with Levene's test. For normally distributed variables, differences between age and sex groups were analysed using independent-samples t-tests; for non-normally distributed variables, the Mann–Whitney *U* test was applied. Paired comparisons between native and reconstructed values were performed using paired t-tests for normally distributed data and Wilcoxon signed-rank tests for non-normally distributed data. Categorical variables were compared using Fisher's exact test. A p-value <0.05 was considered statistically significant. All analyses were performed using SPSS v23 (IBM, IL).

## 3 Results

### 3.1 Study population and comparative approach

The sample size of middle-aged females available for analysis (n = 4) was deemed insufficient for analysis. Consequently, we assessed the effect of gender exclusively within the elderly cohort and evaluated the effect of age by comparing middle-aged and very elderly male subjects.

### 3.2 Implant selection

**Fig. 2:**
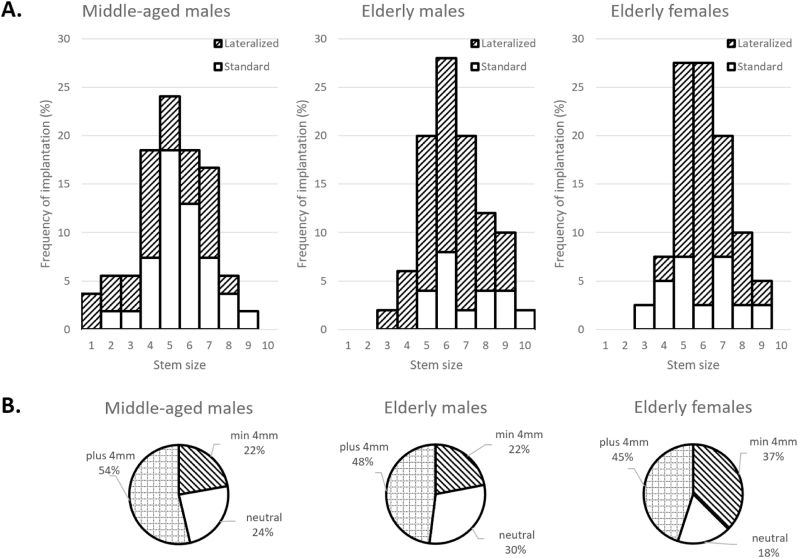
The frequency of component combinations used to achieve optimal reconstruction of the femoral head center in middle-aged males, very elderly males, and very elderly females: (A) stem size and type (standard vs. lateralized) and (B) modular head correction.

Middle-aged males received significantly smaller stems than very elderly males (mean size 5.1 vs. 6.4; p < 0.001). No significant size difference was observed between very elderly males and females (6.4 vs. 6.1; p = 0.204) (median sizes: 5, 6, and 6, respectively; Fig. 2).

Very elderly males had a 3.6° lower NSA and 4.3 mm greater ML-offset compared to middle-aged males; no sex-based differences were found in the elderly group (Table 1). Accordingly, optimal reconstruction using a lateralized stem was more frequently observed in very elderly than in middle-aged males (76.0 % vs. 44.4 %; OR 3.95, 95 % CI 1.71–9.17; p = 0.001), with similar rates between very elderly males and females (76.0 % vs. 67.5 %; OR 1.52, 95 % CI 0.60–3.85; p = 0.478) (Fig. 2). In all groups, the +4 mm modular head was most frequently used (Fig. 2).

**Table 1:** Parameters describing the native and post-reconstruction morphology of the proximal femur per studied group.

	Native Mean ± SD	Native Range	p-value^a^	Post-reconstruction Mean ± SD	Post-reconstruction Range	p-value^b^	Mean difference pre- versus postoperative (p-value^c^)	Proportion of cases showing an increase post-reconstruction

**NSA [°]**								
Middle-aged males	129.5 ± 5.1	118.4–138.1	**<0.001**	127.2 ± 4.0	120.7–132.8	**0.004**	−2.3 (**p=0.001**)	33.3 %
Very elderly males	125.9 ± 5.0	116.3–137.5	N/A	124.7 ± 3.6	121.0–132.3	N/A	NS (p = 0.141)	44.0 %
Very elderly females	124.2 ± 6.0	110.9–140.3	0.152	125.3 ± 4.0	119.6–132.1	0.858	NS (p = 0.273)	52.5 %

**ML-offset [mm]**								
Middle-aged males	42.1 ± 5.4	33.3–54.9	**<0.001**	41.1 ± 4.5	35.1–51.5	**<0.001**	−1.0 (**p=0.001**)	29.6 %
Very elderly males	46.4 ± 5.8	33.4–59.5	N/A	44.8 ± 4.5	35.5–55.2	N/A	−1.5 (**p<0.001**)	24.0 %
Very elderly females	44.2 ± 5.4	26.4–53.5	0.070	43.4 ± 5.1	31.0–54.7	0.205	−0.8 (**p=0.033**)	37.5 %

**FNAA [°]**								
Middle-aged males	9.6 ± 6.1	0.2–24.7	0.639	13.6 ± 7.0	0.3–34.0	0.315	+4.0 (**p<0.001**)	90.7 %
Very elderly males	9.0 ± 5.1	1.1–21.8	N/A	12.3 ± 5.8	1.3–25.5	N/A	+3.3 (**p<0.001**)	84.0 %
Very elderly females	11.2 ± 6.1	1.0–23.4	0.086	14.3 ± 6.5	1.5–25.2	0.140	+3.1 (**p<0.001**)	75.0 %

SD, standard deviation; N/A, not applicable; NSA, neck-shaft angle; ML-offset, mediolateral-offset; FNAA, femoral neck anteversion angle.

P-values that are considered statistically significant (i.e., <0.05) are displayed bold. NS, not significant.

a Native value vs native value for very elderly males.

b Post-reconstruction value vs post-reconstruction value for very elderly males.

c Pre-vs post-reconstruction value.

### 3.3 Accuracy of FHC reconstruction

The mean absolute discrepancy between FHC_native_ and FHC_reconstructed_ of the total cohort was 3.2 mm (±1.8 mm, range: 0.3 mm–10.7 mm), with no statistically significant differences observed between very elderly males and middle-aged males (p = 0.078), or between very elderly males and females (p = 0.592) (Table 2). In 92.4 % of cases, all three dimensions were reconstructed within 5 mm. The odds of inadequate reconstruction in at least one dimension were not significantly higher in very elderly males compared to middle-aged males (OR 3.55, 95 % CI 0.68 to 18.46, p = 0.150), or compared to very elderly females (OR 1.68, 95 % CI 0.38 to 7.19, p = 0.726).

**Table 2:** Accuracy of femoral head center restoration achieved per group with respect to absolute deviation and dimension-specific deviations, described as head height reconstruction, mediolateral offset reconstruction, and anterior offset reconstruction.

	**Qualification of accuracy [n (%)]**				**Quantification of accuracy**	
	**Acceptable**		**Poor**		**Mean** ± **SD**‡ **[mm]**	**Range [mm]**
**Absolute reconstruction**	**<5 mm**		**≥5 mm**			

Total (n = 144)	133 (92.4 %)		11 (7.6 %)		3.2 ± 1.8	0.3 to 10.7
Very elderly males (n = 50)	44 (88.0 %)		6 (12.0 %)		3.6 ± 2.0	0.6 to 10.7
Very elderly females (n = 40)	37 (92.5 %)		3 (7.5 %)		3.2 ± 1.8	0.3 to 7.4
Middle-aged males (n = 54)	52 (96.3 %)		2 (3.7 %)		2.8 ± 1.6	0.4 to 8.8


**Dimension-Specific Deviations**	**<2.5 mm**	**< 5 mm**	**≥5 mm**	**≥10 mm**		

**Head height**						
Very elderly males (n = 50)	28 (56.0 %)	49 (98.0 %)	1 (2.0 %)	0 (0.0 %)	1.1 ± 2.6	−5.0 to 5.0
Very elderly females (n = 40)	25 (62.5 %)	39 (97.5 %)	1 (2.5 %)	0 (0.0 %)	0.7 ± 2.4	−5.0 to 4.5
Middle-aged males (n = 54)	40 (74.1 %)	52 (96.3 %)	2 (3.7 %)	0 (0.0 %)	−0.5 ± 2.1	−5.0 to 7.1

**Mediolateral offset**						
Very elderly males (n = 50)	35 (70.0 %)	45 (90.0 %)	5 (10.0 %)	0 (0.0 %)	−1.5 ± 2.4	−9.4 to 2.31
Very elderly females (n = 40)	28 (70.0 %)	38 (95.0 %)	2 (5.0 %)	0 (0.0 %)	−0.8 ± 2.2	−5.2 to 4.5
Middle-aged males (n = 54)	41 (75.9 %)	53 (98.1 %)	1 (1.9 %)	0 (0.0 %)	−1.0 ± 2.1	−6.2 to 3.6

**Anterior offset**						
Very elderly males (n = 50)	49 (98.0 %)	50 (100.0 %)	0 (0.0 %)	0 (0.0 %)	−0.5 ± 1.2	−5.0 to 2.3
Very elderly females (n = 40)	36 (90.0 %)	39 (97.5 %)	1 (2.5 %)	0 (0.0 %)	−0.3 ± 1.6	−5.7 to 1.9
Middle-aged males (n = 54)	51 (94.4 %)	54 (100.0 %)	0 (0.0 %)	0 (0.0 %)	−0.3 ± 1.2	−3.3 to 2.6

SD, standard deviation; ‡ Positive values for the dimenstion-specific deviations represent a postoperative increase in head height, mediolateral offset and anterior offset respectively.

**Fig. 3:**
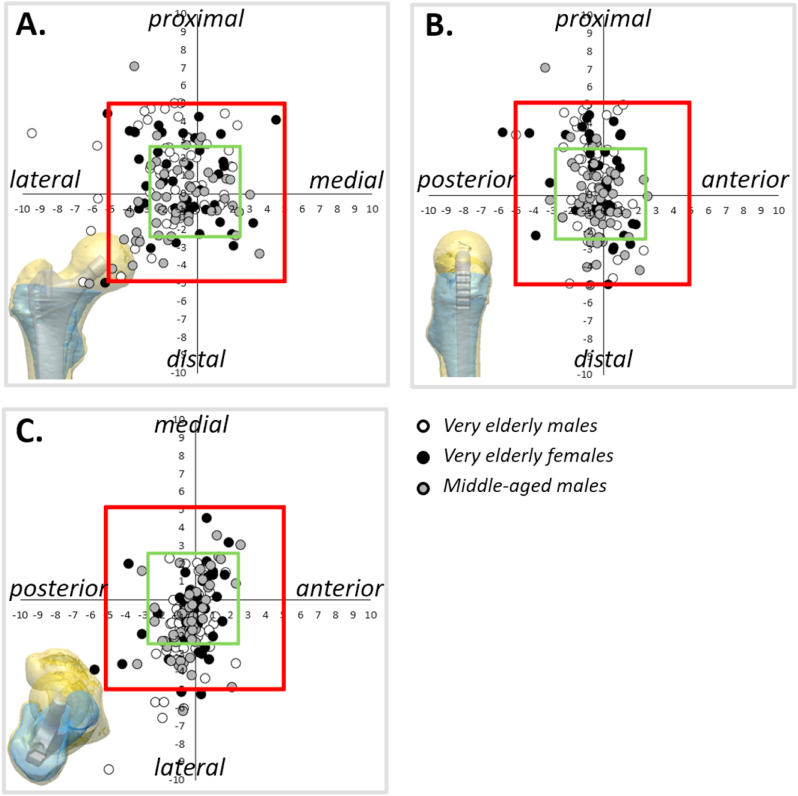
Accuracy of femoral head center reconstruction in three dimensions. Scatter plot of the reconstructed femoral head centers for all subjects in the (A) coronal, (B) sagittal and (C) transversal plane. The origin of the Cartesian coordinate plane represents the location of the native femoral head center and each dot represents the best reconstruction of the femoral head center possible after implanting the femoral component in a subject. The green square represents reconstruction within 2.5 mm discrepancy in each direction of that particular plane, while the red square represents 5 mm discrepancy.

**Fig. 4:**
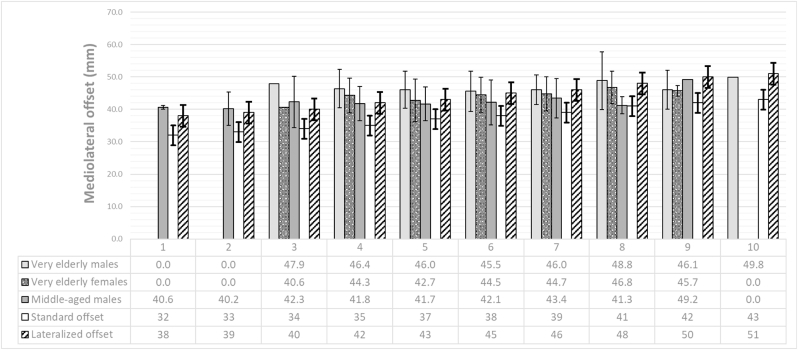
Mediolateral offset reconstruction per stem size. For each implanted stem size, the mean (± standard deviation) native mediolateral offset per studied population and the mediolateral offset ± 4 mm head correction of the particular stem size are shown.

A ≥5 mm deviation was observed in head height in 4 subjects (2.8 %), ML-offset in 8 subjects (5.6 %), and anterior offset in 1 subject (0.7 %) (Table 2, Fig. 3). A slight reduction in ML-offset was observed in the majority of middle-aged males (70.4 %), and very elderly males and females (76.0 % and 62.5 % respectively) (Table 1). Accordingly, it was observed that for each implanted stem size, the mean native ML-offset of each studied population exceeded the standard stem offset (without head correction) for that specific size (Fig. 4). To achieve anterior offset reconstruction, the FNAA increased in 90.7 % of middle-aged males, 84.0 % of very elderly males, and 75.0 % of very elderly females, by an average of 4.0°, 3.3°, and 3.1°, respectively (Table 1).

### 3.4 Mode of failure to accurately reconstruct the FHC

In 11 cases (7.6 %), FHC reconstruction deviated ≥5 mm in at least one direction; their underlying mechanisms were analysed (Table 3, Fig. 3). A recurring failure pattern was noted in five very elderly males: despite using a lateralized stem with a +4 mm head, ML-offset was not adequately restored (absolute decrease: 5.7–9.4 mm), while head height and anterior offset were reconstructed within 5 mm. These patients had low NSA (mean 120.9° ± 3.2°, range: 118.5°–126.4°) and large ML-offset (mean 54.2 mm ± 3.1 mm, range: 51.6–59.5 mm), compared to cohort averages (Table 1). In these morphologies, lateralized stems of size 4 to 6 with +4 mm heads were insufficient to restore ML-offset (Fig. 4). Two additional cases (one middle-aged male, one elderly female) showed combined ML-offset and head height under-reconstruction of ≥5 mm. Both had low NSA (120.2° and 118.2°) and high ML-offset (48.7 mm and 50.3 mm) (Table 1). Although lateralized stems with +4 mm heads were used (sizes 4 and 5), they failed to restore ML-offset and head height. Upsizing the stem led to premature endomedullary engagement and excessive increase of the head height due to proximal positioning of the implant.

**Table 3:** Mechanisms of failure in the 11 cases with reconstruction accuracy deviations ≥5 mm in at least one direction.

**Head height**

**Very elderly males**
- Case 1: A 5.0 mm increase in head height was observed. Native NSA was 127.1°, and native ML-offset was 39.9 mm. A normal stem size 9 with −4 mm head was used, in other directions deviations were <5 mm. A size smaller (size 8) would position the implant too low, even with a standard stem and +4 mm head, leading to a less optimal reconstruction.
**Very elderly females**
- Case 2: A 5.0 mm decrease in head height was observed together with a 5.2 mm decrease in ML-offset. Native NSA was 118.2° and native ML-offset was 50.3 mm, but stem size 5 lateral with a +4 mm head did not achieve the required height nor ML-offset, resulting in an absolute deviation from the native position of 5.2 mm. Attempting a larger size (6) resulted in excessive leg lengthening. To achieve the desired ML-offset, that configuration (lateral stem with +4 mm head) would have been optimal, albeit with a 9.2 mm increase in leg length.
**Middle-aged males**
- Case 3: A 7.1 mm increase in head height was observed. Native NSA was 121.2° and native ML-offset was 49.5 mm. Stem size 4 lateral with a +4 mm head was used. In other directions, deviations were <5 mm. This configuration presented a mismatch: size 4 already resulted in a 7.1 mm increase in head height while leaving a 3.6 mm deficit in ML-offset. Choosing a smaller size to reduce leg lengthening would further exacerbate the ML-offset deficiency and result in an even less optimal reconstruction.
- Case 4: A 5.0 mm decrease in head height was observed together with a 6.2 mm decrease in ML-offset. Native NSA was 120.2° and native ML-offset was 48.7 mm, but the stem size 4 lateral with a +4 mm head did not achieve the required height nor ML-offset, resulting in an absolute deviation from the native position of 7.4 mm. Attempting a larger size would have led to a significant overestimation of head height. Even with the lateral configuration and a +4 mm head, the ML-offset remains insufficient, while leading to a increase in leg length and a 10.4 mm absolute deviation from the native FHC.


**ML-offset**

**Very elderly males**
Each of the following 5 cases presents a comparable mechanism of failure, where the selected lateral stem with a +4 mm head fails to achieve the required ML-offset, with the absolute decrease ranging from 5.7 mm to 9.4 mm (mean: 6.7 mm) while accurately (i.e. <5 mm) restoring head height and anterior offset:
- Case 5: A decrease in ML-offset of 6.5 mm. Native NSA was 118.5° and native ML-offset was 53.2 mm, but the stem size of 4 lateral with a +4 mm head did not reach the required ML-offset. Discrepancies in other directions were below 5 mm, with an absolute deviation from the native position of 7.6 mm.
- Case 6: A decrease in ML-offset of 5.7 mm. Native NSA was 119.7° and native ML-offset was 51.6 mm, but the stem size of 5 lateral with a +4 mm head did not meet the required ML-offset. Discrepancies in other directions were less than 5 mm, resulting in an absolute deviation of 6.6 mm from the native position.
- Case 7: A decrease in ML-offset of 5.7 mm. Native NSA was 120.3° and native ML-offset was 52.6 mm, but the stem size of 5 lateral with a +4 mm head failed to achieve the required ML-offset. Other directional discrepancies remained below 5 mm, with an absolute deviation of 5.7 mm.
- Case 8: A decrease in ML-offset of 6.0 mm. Native NSA was 126.4° and native ML-offset was 53.9 mm, but the stem size of 5 lateral with a +4 mm head did not meet the required ML-offset. Discrepancies in other directions were below 5 mm, with an absolute deviation of 6.2 mm from the native position.
- Case 9: A decrease in ML-offset of 9.4 mm. Native NSA was 119.4° and native ML-offset was 59.5 mm, but the stem size of 6 lateral with a +4 mm head did not achieve the necessary ML-offset. Discrepancies in other directions were less than 5 mm, with an absolute deviation of 10.7 mm from the native position.
**Very elderly females**
- See ‘Case 2’ (underestimation of both head height and ML-offset)
- Case 10: A decrease in ML-offset of 5.1 mm. Native NSA was 124.4° and native ML-offset was 53.5 mm, but the stem size 7 lateral without head correction did not reach the required ML-offset. There was also a 4.5 mm increase in head height (<5 mm). If a +4 mm head had been used, the absolute deviation would have been larger; as it stands, the current configuration yields an absolute deviation of 7.2 mm, representing the optimal reconstruction in this case.
**Middle-aged males**
See ‘Case 4’ (underestimation of both head height and ML-offset)


**Anterior offset**

**Very elderly males**
None
**Very elderly females**
- Case 11: The anterior offset decreased by 5.7 mm; the stem was implanted with a preoperative FNAA of 15.4°, which was increased by only 1° to achieve an ML-offset reconstruction within 5 mm using a size 5 lateralized stem with a +4-mm head. Native NSA was 116.1° and ML-offset 49.6 mm, both the ML-offset and head height were reconstructed within 5 mm.
**Middle-aged males**
None

## 4 Discussion

This study demonstrates that the cementless SL-PLUS™ stem enables anatomically acceptable FHC reconstruction in the vast majority (92.4 %) of both very elderly and middle-aged femora, with no significant differences in reconstruction accuracy at the group level when standard and lateralized stems, along with modular heads of -4 mm, 0 mm, and +4 mm, were used. As hypothesized, we observed the following: (1) Very elderly patients required significantly larger stem sizes, aligning with known age-related femoral canal expansion^11^^,^^12^^,^^15^, ^16^, ^17^ and previously reported correlations between age and implanted cementless stem size.^18^, ^19^, ^20^, ^21^ (2) Optimal reconstruction in very elderly subjects required the lateralized stem variant more frequently than in younger subjects. This is consistent with previously reported varus positioning of the native FHC—characterized by a smaller NSA and higher ML-offset —in older populations.^10^, ^11^, ^12^^,^^14^^,^^41^ The lateralized stem (NSA 123° instead of 131°) with higher ML-offset was optimal in most very elderly males and females (76.0 % and 67.5 %, respectively), typically preserving the NSA postoperatively (Table 1). (3) A failure to adequately restore ML-offset within 5 mm was particularly observed as a mode of failure and mainly in very elderly males with low native NSA and high ML-offset (mean 120.9° and 54.2 mm, respectively), where the implanted stem size resulted in an ML-offset that was on average 6.7 mm less than the native value. This suggests that the specific femoral morphology most commonly seen in this subgroup may exceed the reconstructive capacity of the studied femoral component, despite its overall adequacy at the population level.

A similar stem was studied clinically by Müller et al.,^23^ who studied postoperative changes in proximal femoral anatomical parameters in 44 patients (mean age 65.3 years) following THA with the cementless Zweymüller-Alloclassic® stem (Zimmer Biomet, Warsaw, IN, USA), which shares key features with the design we studied.^35^^,^^37^^,^^42^ In their cohort, preoperative ML-offset and NSA were 39.7 mm and 128.8°,^23^ respectively—2.4 mm and 0.7° lower than those in our middle-aged males (Table 1). Postoperatively, both increased (by 2 mm and 2.8°), along with a leg length increase of 1.3 mm, yielding ML-offset values nearly identical to ours (41.7 mm vs. 41.1 mm), and a higher NSA due to exclusive use of the 131° CCD variant.^23^ An ML-offset increase of approximately 4.5 mm was observed in 70 % of the cases,^23^ whereas we observed a minimal decrease of 0.8–1.5 mm on average (Table 1). FNAA decreased from 24.9° to 7.4°, targeting 10° intraoperatively,^23^ while we observed FNAA increases in most cases, reflecting our focus on native FHC restoration rather than targeting a fixed FNAA. Müller et al. found no association between clinical outcomes and changes in NSA or FNAA in their cohort.^23^

Accurate biomechanical reconstruction is a key goal in THA, and minimizing FHC deviations within 5 mm is often considered a threshold for optimal results.^6^, ^7^, ^8^, ^9^^,^^27^ However, slight increases in ML-offset or head height are preferred over reductions. Cassidy et al. reported worse WOMAC scores in patients with ML-offset reductions >5 mm compared to adequate reconstruction or a slight increase.^8^ Similarly, Inmann et al. found optimal postoperative Harris Hip Score improvements in patients where leg length was reconstructed within 5 mm combined with an accurate to slightly increased general offset (GO; sum of ML-offset and acetabular offset).^7^ Three-dimensional gait analysis 12 months after THA revealed normalized walking speed and hip ROM in patients where leg length and ML-offset were reconstructed within 5 mm,^9^ while Sariali et al. observed gait alternations in patients with a decreased ML-offset of ≥15 % (equating to 6.3–7.0 mm in our cohorts).^4^ Vorimore et al. found that Oxford Hip Score improvement was higher in patients with a reconstructed GO and ML-offset within 5 mm compared to cases where acetabular offset had been compensated for by an increase in ML-offset.^6^ They also observed that optimal clinical improvement was found in subjects where GO and leg length were reconstructed even within 2.5 mm. Although such precision was rare in their study (10 % of cases),^6^ our study showed the ability for absolute reconstruction within 2 mm in 22.0 %–33.3 % of our investigated populations.

The high reconstruction accuracy and consistent slight postoperative decrease in ML-offset across groups (Table 1, Fig. 4) contrasts with clinical studies often reporting ML-offset increases in successful cases.^6^^,^^23^^,^^26^^,^^28^^,^^29^ This difference likely reflects our virtual methodology, which aims to investigate the absolute restoration capability of the native FHC instead of obtaining a stable THA. Scheerlink et al. employed a similar virtual technique to evaluate the reconstructive capability of various stem types—including a cementless calcar-guided stem, a cementless straight stem, an undersized cemented stem, and a cemented line-to-line stem—and reported comparable high overall accuracy (93.9 % within 5 mm vs. 92.4 % in our study).^27^ They found head height reconstruction more consistently achievable than ML-offset, with >5 mm ML-offset deviation occurring in 3.1 % of all reconstructions and 2.0 % of the cementless straight stem subgroup. Notably, their cohort had a mean age of 67 years. In our middle-aged male group, a comparable 1.9 % had ≥5 mm ML-offset deviation, while 10 % and 5 % in our very elderly males and females, respectively. The anterior offset reconstruction accuracy was 0.2 mm on average, which is comparable to our study (mean deviation: 0.3 mm–0.5 mm across groups). While they concluded that both cemented and cementless stems can achieve accurate reconstruction in most non-deformed hips, undersized cemented stems with multiple offset options yielded the best results.^27^

Anterior offset and FNAA reconstruction are often underemphasized in THA planning.^43^ We observed adequate anterior offset reconstruction while increasing the FNAA in most middle-aged males, very elderly males, and females (90.7 %, 84.0 %, and 75.0 %, respectively), with mean increases of 4.0°, 3.3°, and 3.1°, respectively. Dennis et al. found that a postoperative increase of 10° in FNAA generally results in a 7 mm increase in anterior offset and an approximately 5° increase in internal leg rotation.^43^ It has been suggested that achieving a balance in tension between anterior and posterior soft-tissue structures accounts for the observed increase in internal rotation.^43^^,^^44^ Achieving the planned FNAA during surgery—despite 3D-CT preoperative planning or robotic assistance—remains challenging in cementless femoral component positioning,^29^^,^^45^, ^46^, ^47^ whereas it is achievable with cemented fixation.^47^ It has even been argued that postoperative FNAA cannot be reliably related to native FNAA, since most modern cementless designs simply follow the metaphyseal twist to a best-fit position during implantation, without allowing for the needed adjustments.^45^^,^^46^ In contrast, such adjustment remains possible with cemented designs.^45^^,^^47^ An increased FNAA may elevate the risk of ischiofemoral impingement.^48^ A ′femur-first' strategy—where the acetabular cup is placed after the cementless femoral component to achieve functional combined anteversion—has been proposed as a potential solution.^46^ The exclusion of the acetabular component from this study may be regarded as a limitation.

Several limitations should be considered when interpreting these results. First, the sample size limits the generalizability of subgroup analyses. In particular, the number of middle-aged females (n = 4) was insufficient for meaningful analysis, restricting the evaluation of age effects to males and sex differences to very elderly subjects. Post hoc power analysis revealed sufficient power to detect differences in stem size and lateral stem use between very elderly and middle-aged males (98 % and 92 %, respectively), but not for differences in reconstruction accuracy (61.1 % for continuous, 35.5 % for dichotomous outcomes). Despite the limited statistical power, we were still able to identify meaningful trends in the mechanisms underlying reconstruction failure. Second, the findings apply exclusively to the SL-PLUS™ stem combined with −4 mm and +4 mm modular heads. Results are therefore not generalizable to other stem designs or modular head options, which may offer greater reconstructive flexibility. Third, although stem implantation was meticulously performed in accordance with the design's principles—and our methodology builds on prior experience^27^^,^^38^, ^39^, ^40^—this study remains an *in-silico* simulation rather than a clinical study. Consequently, reproducibility in clinical practice cannot be assured. Finally, while the objective of striving for the most accurate exact restoration of the FHC_native_ is methodologically sound, it may result in minor decreases in ML-offset or head height, which are suboptimal in clinical reality.^6^^,^^23^^,^^26^^,^^28^^,^^29^

## 5 Conclusions

Despite anatomical variability and the inherent limitations of a single cementless stem design, FHC reconstruction was generally accurate and consistent across groups. Very elderly patients required significantly larger and more lateralized stems, consistent with previous findings of age-related canal widening and increased native ML-offset in this demographic. However, specific femoral morphologies—particularly the combination of low NSA and high ML-offset, predominantly seen in very elderly males—may exceed the stem's reconstructive capacity. Preoperative templating using 2D radiographs or preferably 3D-CT^49^^,^^50^ is essential to identify cases in which adequate FHC reconstruction cannot be achieved with the appropriate stem size. Early recognition of such cases facilitates the selection of suitable alternatives, such as extended modular heads (e.g., +8 mm, +12 mm, or +16 mm), alternative cementless or cemented stem designs, or, in selected cases, a modular or even custom-made stem.

## CRediT authorship contribution statement

H.D. Veldman: Conceptualization, Methodology, Formal analysis, Investigation, Writing – original draft.

I.C. Heyligers: Conceptualization, Methodology, Formal analysis, Investigation, Writing – review & editing.

P.C. Noble: Conceptualization, Methodology, Writing – review & editing.

T.A.E.J. Boymans: Conceptualization, Methodology, Formal analysis, Investigation, Writing – review & editing.

All authors have agreed to be held accountable for the content of the work.

## Guardian patient's consent

Written informed consent was obtained from all patients included in this study.

## Ethical statement

The study was reviewed and approved by our local institutional review board (approval number 07-T-44/IIIb).

## Funding

There was no funding for this project.

## Conflict of interest

One of the authors is a member of the Board of Directors of the International Society for Technology in Arthroplasty (voluntary, non-remunerated position). The authors declare that the research was conducted in the absence of any commercial or financial relationships that could be construed as a potential conflict of interest.
